# Differing Patterns of Selection and Geospatial Genetic Diversity within Two Leading *Plasmodium vivax* Candidate Vaccine Antigens

**DOI:** 10.1371/journal.pntd.0002796

**Published:** 2014-04-17

**Authors:** Christian M. Parobek, Jeffrey A. Bailey, Nicholas J. Hathaway, Duong Socheat, William O. Rogers, Jonathan J. Juliano

**Affiliations:** 1 School of Medicine, University of North Carolina, Chapel Hill, North Carolina, United States of America; 2 Curriculum in Genetics and Molecular Biology, University of North Carolina, Chapel Hill, North Carolina, United States of America; 3 Program in Bioinformatics and Integrative Biology, University of Massachusetts, Worcester, Massachusetts, United States of America; 4 Division of Transfusion Medicine, School of Medicine, University of Massachusetts Medical School, Worcester, Massachusetts, United States of America; 5 School of Medicine, University of Massachusetts, Worcester, Massachusetts, United States of America; 6 National Malaria Center, Phnom Penh, Cambodia; 7 United States Navy, Naval Medical Research Unit #2, Phnom Penh, Cambodia; 8 Division of Infectious Diseases, University of North Carolina School of Medicine, Chapel Hill, North Carolina, United States of America; Federal University of São Paulo, Brazil

## Abstract

Although *Plasmodium vivax* is a leading cause of malaria around the world, only a handful of vivax antigens are being studied for vaccine development. Here, we investigated genetic signatures of selection and geospatial genetic diversity of two leading vivax vaccine antigens – *Plasmodium vivax* merozoite surface protein 1 (*pvmsp-1*) and *Plasmodium vivax* circumsporozoite protein (*pvcsp*). Using scalable next-generation sequencing, we deep-sequenced amplicons of the 42 kDa region of *pvmsp-1* (n = 44) and the complete gene of *pvcsp* (n = 47) from Cambodian isolates. These sequences were then compared with global parasite populations obtained from GenBank. Using a combination of statistical and phylogenetic methods to assess for selection and population structure, we found strong evidence of balancing selection in the 42 kDa region of *pvmsp-1*, which varied significantly over the length of the gene, consistent with immune-mediated selection. In *pvcsp*, the highly variable central repeat region also showed patterns consistent with immune selection, which were lacking outside the repeat. The patterns of selection seen in both genes differed from their *P. falciparum* orthologs. In addition, we found that, similar to merozoite antigens from *P. falciparum* malaria, genetic diversity of *pvmsp-1* sequences showed no geographic clustering, while the non-merozoite antigen, *pvcsp*, showed strong geographic clustering. These findings suggest that while immune selection may act on both vivax vaccine candidate antigens, the geographic distribution of genetic variability differs greatly between these two genes. The selective forces driving this diversification could lead to antigen escape and vaccine failure. Better understanding the geographic distribution of genetic variability in vaccine candidate antigens will be key to designing and implementing efficacious vaccines.

## Author Summary

*Plasmodium vivax* causes tens of millions of malaria cases each year. Although some vaccines against *P. vivax* are being developed, little is known about the geospatial genetic diversity and selective constraints of the parasite surface antigens that these vaccines target. In order to create vaccines that are both efficacious and useful in diverse regions of the world, the strain diversity of these potential vaccine targets must be well understood. Specifically, we must understand whether and how the human immune system develops immunity against these antigens as well as understanding whether these antigens are similar in geographically diverse parasite populations. Here, using next-generation sequencing and population-genetic analyses, we found evidence of likely immune selection in specific regions of two leading vivax vaccine candidate antigens, PvMSP-1 and PvCSP. At the *pvmsp-1* locus, we also found more genetic variability within populations than between populations, with some DNA sequences from geographically diverse populations being highly similar. In contrast, *pvcsp* sequences from geographically diverse populations are very distinct from one another, with specific sequence patterns occurring in certain geographic regions. Our findings provide new insights into the geographic genetic diversity of these two antigens and can help inform the development of effective *P. vivax* vaccines.

## Introduction

*Plasmodium vivax* causes 80 to 300 million infections per year and over 2.5 billion people remain at risk of infection despite malaria elimination efforts [1]. Now, concern over *P. vivax* is growing due to reports of increasingly severe disease [2], emerging chloroquine resistance [3], and multi-drug resistance [4]. Ultimately, an effective vaccine will be important for controlling *P. vivax* malaria [5]. The fact that humans naturally develop partial immunity to *P. vivax* and *P. falciparum* lends hope for effective vaccines against these parasites; however, because the majority of global malaria research funding targets *P. falciparum* [6], [7], only a handful of *P. vivax* antigens are currently being considered for vaccine development [8]. Among these are *P. vivax* merozoite surface protein 1 (*pvmsp-1*) and circumsporozoite protein (*pvcsp*).

**Figure 1:**
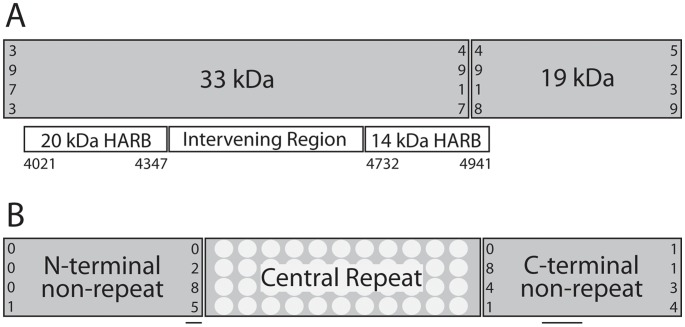
Protein domains and immunologically-relevant regions of *pvmsp-1* 42 kDa region and *pvcsp*. For both genes, numbers indicate coordinates according to the Sal1 reference genes. Sequences for *pvmsp-1* (PVX_099980) and pvcsp (PVX_119355) were accessed August 14, 2012 from PlasmoDB.org. (**A**) The *pvmsp-1* 42 kDa region is composed of two primary subunits – a 33 kDa and a 19 kDa subunit. Other sub-regions, including the 20 kDa and 14 kDa HARBs have been previously defined and studied. Here, we define the region between the HARBs as the “intervening region.” (**B**) The *pvcsp* gene is composed of three regions – an N-terminal non-repeat region, a central repeat region, and a C-terminal non-repeat region. The central repeat region consists of two major nonapeptide repeat types, termed VK210 and VK247. Approximate locations of *pvcsp* regions I and II are noted with horizontal lines in the N- and C-terminal non-repeat regions, respectively.

PvMSP-1, an erythrocytic vaccine candidate, plays an important role in reticulocyte invasion [9]. Its C-terminus contains a 42 kDa region, which is processed into 33 and 19 kDa fragments (**Figure 1A**). The 33 kDa fragment contains two high-affinity reticulocyte binding clusters (HARBs) (20 kDa and 14 kDa), and antibodies against the HARBs confer protection in monkeys [10]. In humans, antibodies to the 42 kDa region have also been associated with clinical protection, making this region an attractive vaccine candidate [11]–[14]. Another vivax protein, PvCSP, is a pre-erythrocytic vaccine candidate and is critical in sporozoite motility and hepatocyte invasion [15]. *P. vivax* circumsporozoite protein has an immunogenic central repeat, consisting of two major types of nonapeptide repeats (VK210 and VK247 – there is also a rarer repeat type termed *vivax-like*) flanked by highly conserved 5′ and 3′ regions (**Figure 1B**). The *P. falciparum* ortholog of *pvcsp*, as formulated in RTS,S, is the most advanced *P. falciparum* vaccine candidate to date, showing modest efficacy at one year interim analysis in a Phase III trial [16].

Despite this knowledge of PvMSP-1 and PvCSP, little is known about the geospatial genetic diversity of these antigens. Variation in these antigens may become a mechanism of vaccine resistance if strain-specific immunity is important in protection, as has been seen in some *P. falciparum* vaccine candidates [17]. Vaccine trials of *P. falciparum* AMA1 and MSP2 as well as genetic crosses using *P. chabaudi* underscore the importance of strain-specific immunity as a determinant of outcome [18]–[21]. Additionally, despite initial evidence that strain-specific immunity may not impact RTS,S efficacy [22]–[25], the incomplete protection afforded by the RTS,S vaccine in Phase II and III trials [16], [26], [27] has prompted a careful examination of strain-specific responses to this vaccine. Thus, as momentum grows for field trials of *P. vivax* vaccine antigens, carefully designed population genetic studies of *P. vivax* vaccine candidates will be key to assess the need for multivalent vaccine formulations.

To better understand the selective forces on, and geospatial genetic diversity associated with *pvmsp-1* and *pvcsp*, we used the Illumina sequencing platform to determine haplotypes for 42 kDa region of *pvmsp-1* (n = 44) and we used the PacBio and Illumina platforms to sequence the complete *pvcsp* gene (n = 47) from Cambodian isolates [28]. To dissect the immune selection acting on these regions, we studied these sequences using population genetic tests of selection and models of tandem repeat evolution. To evaluate the global genetic diversity of *pvmsp-1* and *pvcsp*, we extracted worldwide *pvmsp-1* and *pvcsp* sequence data available in GenBank (n = 238 for *pvmsp-1* and n = 412 for *pvcsp*) (**Figure S1**), and studied our sequence data alongside the sequences from GenBank *msp-1*. Finally, we compare the performance of Illumina and PacBio sequencing to traditional Sanger sequencing, and discuss the potential and challenges of next-generation sequencing for population genetic studies of malaria parasite antigens.

## Methods

### Parasite isolates

Clinical samples from a previous study were used for this study [29]. Written informed consent was acquired from each individual and the study was approved by the IRB at University of North Carolina, the IRB of the Naval Medical Research Unit #2, Jakarta, Indonesia, and the Cambodian National Ethical Committee for Health Research. Briefly, blood spots were collected from 109 patients with uncomplicated vivax malaria, presenting to a clinic in Chumkiri, Cambodia during 2006–07. We selected 48 subjects with a multiplicity of infection (MOI) of one (n = 20) or two (n = 28) for sequencing. MOI was determined by heteroduplex tracking assay (HTA) [28], [30]. Briefly, in an HTA, radiolabeled DNA probes are annealed to genomic DNA and drawn through a non-denaturing gel matrix. The number of bands observed represents the number of conformation differences present among heteroduplexes, and is a proxy for the number of infection clones (MOI). Details of the method have been published elsewhere [31].

### Amplification of *pvmsp-1* and *pvcsp*

The *pvmsp-1* 42 kDa region (nucleotides 3973–5239 of Sal1 PVX_099980, www.PlasmoDB.org) was amplified using primers F: 5′-CAG GAC TAC GCC GAG GAC TA-3′ and R: 5′-GGA GGA AAA GCA ACA TGA GC-3′ and an Eppendorf Mastercycler (Eppendorf, Hauppauge, NY) in 50 µL reactions containing 5 µL 10× Qiagen Hotstar Master Mix (Qiagen, Valencia, CA), 0.25 µL Qiagen Hotstar *Taq*, 300 nM forward primer, 300 nM reverse primer, 1 µL 10 mM dNTPs, and 5 µL 5–10 mM template. Cycling conditions were: 95°C×15 m; 35 cycles of 95°C×45 s, 55°C×45 s, 72°C×3 m; and 72°C×10 m. The *pvcsp* gene (PVX_119355) was performed by nested PCR. The outer step used primers F: 5′-GGC AAA CTC ACA AAC ATC CA-3′ and R: 5′-TGC GTA AGC GCA TAA TGT GT-3′. Reactions were as above except for 600 nM forward primer, 600 nM reverse primer, 1 µL 10 mM dNTPs, 5 µL 5–10 mM template, 6 µL of 25 mM MgCl_2_, and 28.75 µL H2O. Cycling conditions were: 95°C×15 m; 25 cycles of 95°C×45 s, 45°C×45 s, 72°C×3 m; and 72°C×10 m. The inner step used 600 nM of each of the primers F: 5′-AAA CAG CCA AAG GCC TAC AA-3′ and R: 5′-GAC GCC GAA AAT ATT GGA TG-3′ using 5–10 µL of the initial amplification. The cycling conditions were: 95°C×15 m; 25 cycles of 95°C×45 s, 54°C×45 s, 72°C×3 m; and 72°C×10 m.

### Amplicon sequencing and sequence determination

*pvmsp-1* and *pvcsp* amplicons were fragmented by acoustic shearing (Covaris, Woburn, MA) using the following settings: 10% duty cycle, 5.0 intensity, 200 cycles per burst, and frequency sweeping mode. Forty-eight barcoded libraries were prepared using the NEXTflex multiplex library kit (Bioo Scientific, Austin, Texas), each containing the pooled *pvmsp-1* and *pvcsp* amplicons from one patient. Libraries were sequenced on the Illumina HiSeq2000, using the paired-end 100 base pair chemistry (Illumina, San Diego, CA).

We used Lasergene SeqMan NGen v.3.1.1 (DNASTAR, Madison, WI) to assemble *pvmsp-1* short reads *de novo* and to determine SNP frequency within each assembly. For purposes of comparison and confirmation, we re-sequenced 9 *pvmsp-1* amplicons with differing MAFs: 3 samples with all MAFs>90%; 3 samples with all MAFs between 60% and 90%; 3 samples with MAF<60% for at least one SNP. Sanger-sequence haplotypes were compared to predicted Illumina haplotypes. Based on these comparisons, only predicted *pvmsp-1* haplotypes with MAF>60% at all polymorphic sites were used in our analysis.

In addition to Illumina sequencing, *pvcsp* amplicons were sequenced using PacBio Circular Consensus Sequencing (Pacific Biosciences, Menlo Park, CA). One PacBio SMRT cell produced a total of 12103 reads with a minimum of 3× circular consensus coverage, which were used for this study. These were further filtered, removing truncated reads or reads with errors in the barcode. This left 8430 reads (3979 forward and 4451 reverse). Clustering attempted to minimize false positive haplotypes due to erroneous base calls and PCR slippage within the tandem repeat region. For each sample, haplotypes were created by clustering reads, allowing reads differing only by indels of 1 and 2 bases and low quality mismatches to collapse. Low quality was defined as either a mismatching base Q<30 or any Q<25 within an 11 basepair region centered on the mismatch, as has been applied previously to rigorous SNP discovery from shotgun data [32]. To overcome artifacts of PCR infidelity due to slippage events leading to shortened repeats and false haplotypes, we set a high threshold requiring that co-occurring haplotypes of the same repeat type be at high frequency in order to exclude the low frequency variation/stuttering in the repeat region. Haplotype repeat type was then determined by translation and the most frequent haplotype of each major repeat type (VK210 and VK247) present was kept >0.5%. Additional haplotypes of major repeat types were kept if they were common (>20%) and thus unlikely to be due simply to low frequency slippage events. In total across all samples 4081 of the 8430 reads clustered contributed to utilized haplotypes.

The long-read haplotypes determined through consensus clustering were used as templates for short-read alignment using Bowtie2 v 2.1.0 [33], with very-sensitive alignment parameters and stringent filtering for Mapping Quality Score and Alignment Score. Final sequence predictions were used for the analyses in this paper and were deposited in GenBank under accession numbers JX461243-JX461285 and KJ173797- KJ173802 for *pvcsp*, and JX461286-JX461333 for *pvmsp-1*.

Rarefaction curves of haplotypes were calculated using EstimateS v9.0. Individual-based curves using sampling without replacement were estimated [34] and extrapolated to 2× the actual sample number [35]. Rarefaction plots were visualized in the R base package (http://cran.us.r-project.org/).

### Acquisition of published sequences for inter-population comparisons

GenBank was queried for population sets published prior to August 1, 2013, which included sequence data for the 42 kDa region of *pvmsp-1* and the whole-gene of *pvcsp*. Sequences from a recent publication [36] were excluded because the isolates were collected over the course of a 12 year period. The authors provide evidence that the haplotype distribution of this population changed substantially over time, making this population inappropriate for our analysis of selection.

### Assessing selection on *pvmsp-1* and *pvcsp*

Population datasets with >25 sequences that were collected over a span of ≤4 years were included for analysis of selection. We used DnaSP v5.1 to perform tests of selection [37]. We calculated polymorphism and Tajima's D across *pvmsp-1* and the *pvcsp* constant regions using a 50 bp sliding window with a 25 bp step size. We also performed 1000 coalescent simulations with recombination to determine a 95% confidence interval and centile for each Tajima's D estimate [38]. To test for long-term selection, we used the McDonald-Kreitman (MK) test [39]. Skew was calculated using Fisher's exact test (two tailed). For the *pvmsp-1* 42 kDa region amplicons reported here and by others, 15 *Plasmodium knowlesi pkmsp-1* isolates from Thailand [40] (Accession Nos. JF837339-JF837353) were used as the interspecies outgroup. Three insertions and deletions occurred in the 42 kDa region of *pvmsp-1* relative to *pkmsp-1*, and were not considered. We could not obtain MK estimates for *pvcsp* sequences due to numerous insertions and deletions relative to *pkcsp*.

For analysis of *pvcsp* repeats, we performed pairwise comparisons of untranslated repeat units within individual *pvcsp* sequences [41]. We calculated skewness and mean nucleotide differences between repeat units, as previously reported [42]. Similar to the methods of Dias et al., 2013, we also calculated dN/dS on the first 1–459 bases of all 32 VK210 repeat regions and the first 1–540 bases of all 15 VK247 repeat regions. This analysis was performed in MEGA5, using the Nei-Gojobori method [43].

### Phylogenetics and statistics to determine population structure

Interpopulation heterogeneity was first assessed using Wright's fixation index (*F*_ST_). Pairwise fixation values between *pvmsp-1* populations were calculated in DnaSP. Site-specific fixation values for pairwise comparisons among Cambodia, NW Thailand, S Thailand, India, and Turkey were generated using the analysis of molecular variance (AMOVA) function within Arlequin v3.11 [44].

Neighbor-joining trees for *pvmsp-1*, *pvcsp* VK210, and *pvcsp* VK247 were drawn using the APE package for R [45]. To generate trees based off *pvmsp-1*, distance calculations between haplotypes were performed in MEGA5 using the maximum composite likelihood method to construct a neighbor-joining tree file. For the *pvcsp* CR, we used MS_Align (v.2.0) [46], [47] to create genetic distance matrices separately comparing both the VK210 and VK247 repeat arrays. MS_Align generates an event-based genetic distance using a model of tandem repeat evolution (expansion, deletion, substitution). Cost parameters for MS_Align were set to 0.1 for amplification or contraction and 5 for repeat insertion or deletion. A pairwise cost table of repeat-to-repeat mutations was created in MEGA5 using the maximum composite likelihood method and used as input for MS_Align [41], [48]. MS_Align output matrices were used by FastME [49], [50] to construct neighbor-joining trees with balanced branch-length estimation.

To cluster geographic groups, we calculated Hudson's nearest-neighbor statistic (S_NN_) [51]. Input was in the form of a pairwise distance matrix between all haplotypes for each phylogeny. For this statistic, highly distant populations have values approaching 1 while panmictic populations have values near 0.5. To test the reproducibility of the geographic clustering predicted by S_NN_, 1000 jackknife samplings were constructed for both *pvmsp-1* and *pvcsp* VK210 and VK247 populations using Fast UniFrac [52]. For each jackknife replicate, 5 individuals, based on the size of the smallest population, were randomly selected from each population and used to redraw trees. Observed splits between geographic populations were quantified and used to assign confidence to predicted geographic clusters. To evaluate potential mutational paths connecting all *pvmsp-1* haplotypes, we constructed a median-joining network using NETWORK v4.6 (Fluxus Engineering, Suffolk, England) [53]. This method expresses multiple plausible evolutionary paths in the form of cycles. A similar analysis was not completed for *pvcsp* due to the variable length of CR haplotypes.

## Results

### *pvmsp-1* sequences

**Figure 2:**
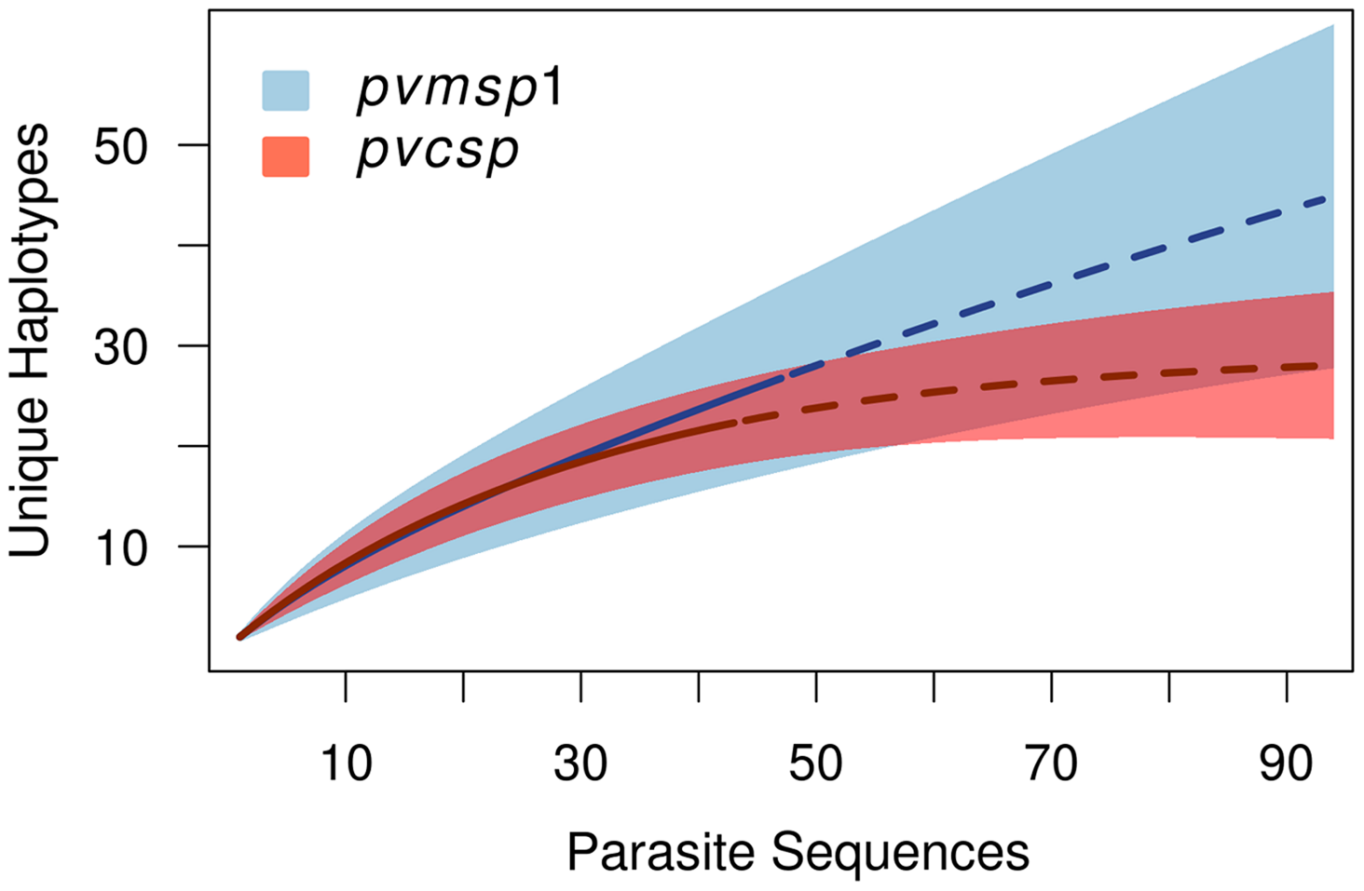
Haplotype rarefaction curves for the Cambodian cohort. Calculated rarefaction curves are depicted by solid blue (*pvmsp-1*) and red (*pvcsp*) lines. Dotted lines represent rarefaction values extrapolated according to the methods of Cowell, et al. The 95% CIs of rarefaction estimates for *pvmsp-1* and *pvcsp* are demarked by light blue and light red shaded areas, respectively.

We Illumina sequenced *pvmsp-1* 42 kDa-fragments (**Figure 1A**) from 48 patients, and compared these to Sanger sequencing data for selected samples. Illumina haplotypes with a major allele frequency of >60% agreed with Sanger haplotypes in every case tested (n = 6). Illumina haplotypes with a major allele frequency of <60% did not consistently agree with Sanger haplotypes (n = 3). Thus, we were able to build 44 complete *pvmsp-1* 42 kDa haplotypes (26 unique haplotypes) with a major allele frequency of >60% at all polymorphic sites (**Table 1**). The average coverage depth for all isolates was >800 reads per base, with all bases having ≥100 reads of coverage. Haplotype accumulation (rarefaction) curves were estimated, and then further extrapolated to show that our sample captured fewer than half the total *pvmsp-1* haplotypes in this region of Cambodia (**Figure 2**). In addition to these isolates, we identified 238 submissions in GenBank [54]–[58] (**Table S1**) containing either the whole-gene or 42 kDa-region sequence information.

**Table 1:** Summary population genetic data for *Plasmodium vivax* antigens.

Country of Origin	*n* 1	*S* 2	*K* 3	*π* 4	*H* 5	*Hd* 6	*Tajima's D*
***pvmsp-1*** **: 42 kDa region**							
Cambodia	44	62	24.8	0.020	26	0.950	2.08*
India	28	64	24.9	0.021	27	0.997	1.32*
NW Thailand	65	62	24.9	0.020	34	0.968	2.42*
S Thailand	67	42	6.46	0.005	5	0.336	−0.986
Turkey	30	33	8.33	0.007	3	0.536	−0.001
***pvcsp*** **: N- and C-terminal non-repeat regions**							
Cambodia	47	-	-	-	24	-	-
N-terminal non-repeat		3	0.971	0.003	3	0.500	0.901
C-terminal non-repeat		2	0.318	0.001	2	0.159	−0.538
Columbia	27	-	-	-	27	-	-
N-terminal non-repeat		2	0.285	0.001	2	0.143	−0.954
C-terminal non-repeat		0	-	-	1	0.000	-

This table includes all population sequence sets which contained sufficient numbers to perform allele-based tests of neutrality. Population sets which included sequence data only for *pvcsp* repeat regions alone are not summarized here.

*p<0.05;

1 number of haplotypes;

2 within-population variant sites;

3 average number of nucleotide differences;

4 nucleotide diversity;

5 number of haplotypes;

6 haplotype diversity.

### Detecting signatures of selection within *pvmsp-1*

**Figure 3:**
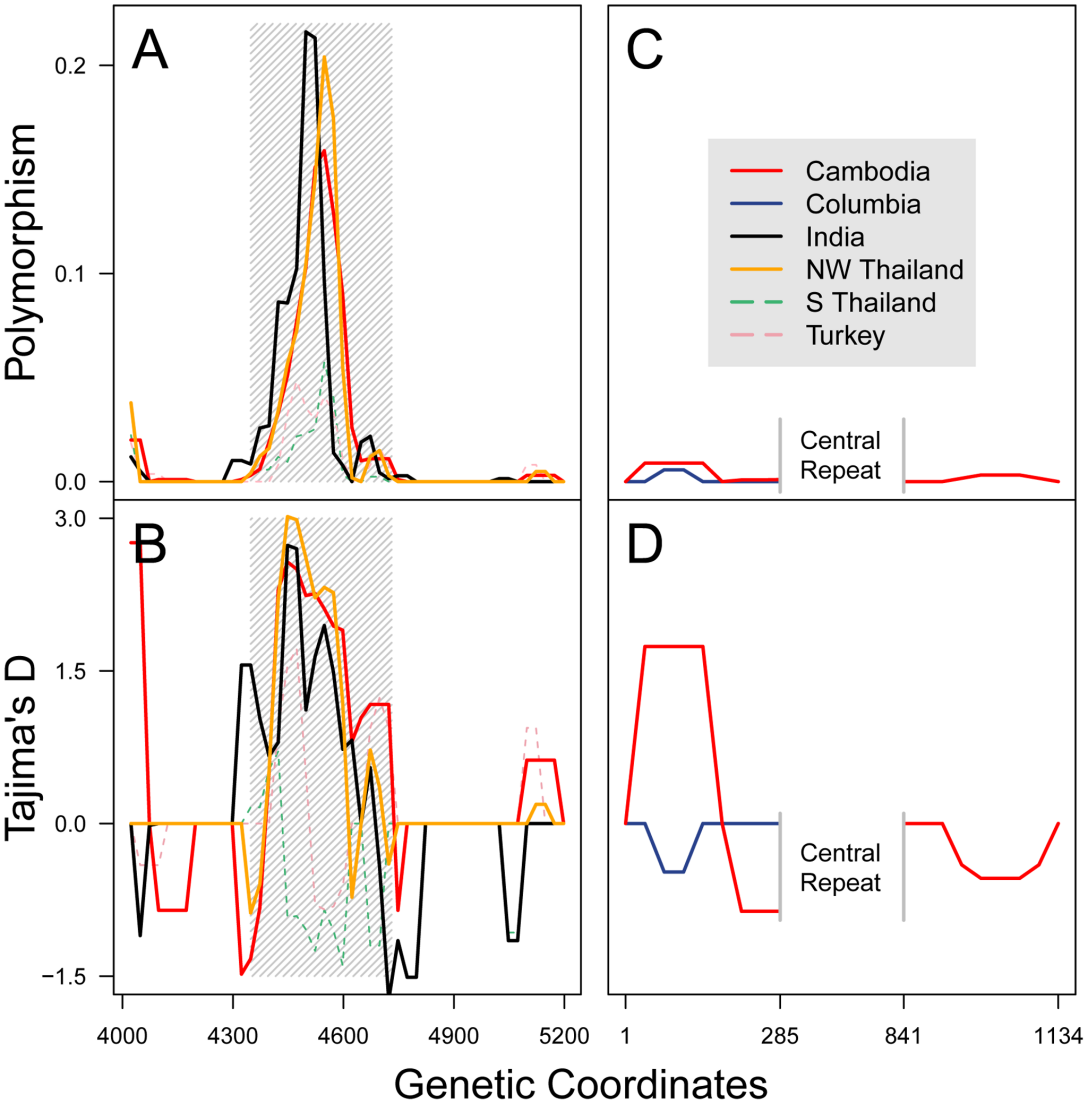
Nucleotide diversity and Tajima's D across the *pvmsp-1* 42 kDa region and the whole *pvcsp* gene. Polymorphism (nucleotide diversity, *π*) (**A**) and Tajima's D (**B**) were calculated across the *pvmsp-1* amplicon for five diverse populations. A sliding window (50 bp window and 25 bp step size) was used to achieve a high resolution analysis. Grey hatches demark the intervening region (nucleotides 4348–4731). For *pvcsp*, N-terminal and C-terminal non-repeat regions were analyzed for nucleotide polymorphism (**C**) and evidence of balancing selection (**D**) using a sliding window. Putatively panmictic populations are marked with a solid line, while populations known to be subject to strong selective forces are marked with dotted lines. All coordinates are based on Sal1 *pvmsp-1* and *pvcsp* reference sequences.

The interaction between human host and the parasite has had a profound impact on the parasite genome, leaving behind characteristic “signatures” of natural selection [59], which are detectable using population genetics approaches to examine sequence diversity. We first assessed nucleotide diversity (**Figure 3A**), and observed a spike of polymorphism in the region between the two HARBs (positions 4348–4731 in the Sal1 reference). We termed this the “intervening region”. To test whether the diversity in the intervening region is due to long-term selection, we used the McDonald-Kreitman (MK) test [39] to compare the ratio of non-synonymous to synonymous nucleotide polymorphisms between the Cambodian *P. vivax* population and a Thai *P. knowlesi* population [40]. We observed a highly elevated MK ratio (*p* = 0.00427) in the intervening region but not in the HARBs (data not shown) or the entire 42 kDa region (*p* = 0.681), suggesting that the intervening region is under long-term selective pressure (**Table 2**).

**Table 2:** McDonald-Kreitman test for selection in *pvmsp-1*.

McDonald-Kreitman Comparisons				
	42 kDa region		42 kDa intervening region	
	Synonymous	Non-synonymous	Synonymous	Non-synonymous
Fixed	96	93	32	28
Polymorphic	34	39	10	31
	*p* = 0.681		*p* = 0.00427	

Evidence for long-term selective pressure on the *pvmsp-1* 42 kDa region and the 42 kDa intervening region was assessed with the McDonald-Kreitman test, using *P. knowlesi msp1* as the outgroup comparator. A Fisher's exact test (two tailed) was used to determine significance.

To determine whether the long-term selective pressure shaping the intervening region is potentially due to human immunity, we assessed balancing selection in this region, as balancing selection within a malaria antigen suggests that the antigen is a target of the human immune system [59]. We applied Tajima's D test of neutrality [60] to five geographically distinct *P. vivax* populations (all populations with n>25, accounting for 190 of 238 available sequences) (**Table 1** **,** **Figure 3B**). In panmictic populations with an uncomplicated demographic history [59], the Tajima's D statistic can indicate whether a nucleotide sequence is under directional (D<0) or balancing selection (D>0). Populations not subjected to recent bottlenecks (i.e. Cambodia, India, and NW Thailand, [54], [58]) demonstrated a significant signature of balancing selection in the *pvmsp-1* 42 kDa region (**Table 1**). This signature occurred specifically in the intervening region (**Figure 3B**), and is consistent with the conclusion that human immunity targets the intervening region.

The three regions of the *pvmsp-1* fragment that are considered vaccine candidates were each assessed for diversity in the Cambodian population [9], [61]. In contrast to the intervening region, the 20 kDa HARB (Sal1 positions 4021–4347) and 14 kDa HARB (Sal1 positions 4732–4941) showed no coding polymorphisms and no evidence of balancing selection, similar to recent reports [61]. The 19 kDa fragment (Sal1 nucleotide positions 4918–5239) also showed limited diversity, with only a K1709E substitution, and no evidence of balancing selection.

### Geospatial genetic diversity at the *pvmsp-1* 42 kDa region

Although the *pvmsp-1* 42 kDa region contains potential vaccine candidates [9], [61], the 42 kD region's global genetic diversity has not been carefully evaluated. To study *pvmsp-1* 42 kDa diversity, we calculated Wright's Fixation index (*F*_ST_) [62] for each pairwise comparison between five diverse populations (**Table 3**). *F*_ST_ values between naturally evolving parasite populations (Cambodia, NW Thailand, and India) approached zero, showing a high degree of genetic similarity, while comparisons with populations that have undergone a recent bottleneck (S Thailand and Turkey) showed a high degree of genetic distance due to their limited number of haplotypes. Similarly, *F*_ST_ values calculated for each variable site demonstrate a high degree of homogeneity in pairwise comparisons between the Cambodia, NW Thailand, and India populations across all sites, and substantial heterogeneity between S Thailand and Turkey across all sites (**Figure S2**). This is evidence that balancing selection maintains a similar range of alleles in the *pvmsp-1* 42 kDa region of multiple geographically diverse naturally evolving *P. vivax* populations.

**Table 3:** Interpopulation *F*-statistics for *pvmsp-1*.

*pvmsp-1* Global *F*_ST_ 0.340				
*pvmsp-1* Pairwise	Cambodia	India	NW Thailand	S Thailand
**India**	0.031			
**NW Thailand**	0.000	0.043		
**S. Thailand**	0.449	0.433	0.366	
**Turkey**	0.361	0.329	0.403	0.796

*F*_ST_ values compare the relatedness of a gene among different populations of the same species. Reported values compare the relatedness of *pvmsp-1* 42 kDa alleles for pairwise comparisons between Cambodia, India, NW Thailand, S Thailand, and Turkey. *F*_ST_ values approaching 0 indicate greater relatedness, while values approaching 1 indicate substantial inter-population variability. Global *F*_ST_ statistic calculated between all *pvmsp-1* populations with n>25 indicates that relatively little genetic distance exists between the sampled populations. However, pairwise comparisons demonstrate that some populations exhibit a high degree of genetic similarity (Cambodia and India, for example) while other populations are more dissimilar (S Thailand and Turkey, for example).

**Figure 4:**
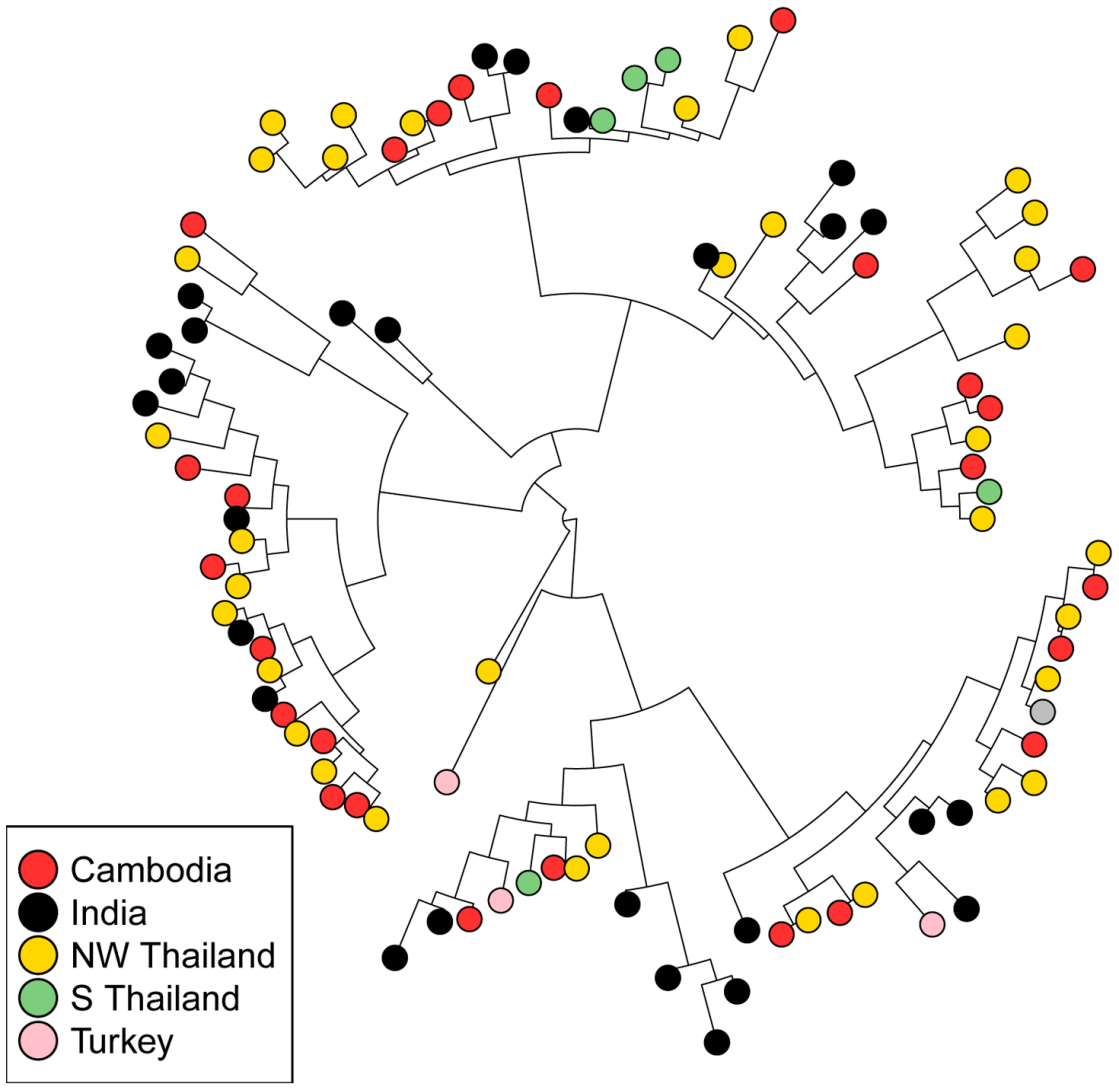
Neighbor-joining tree of 42 kDa regions from *pvmsp-1* isolates. All unique 42*pvmsp-1* population set with n>25 were plotted on a single unrooted, neighbor-joining phylogenetic tree. The Sal1 reference sequence is marked in grey.

**Figure 5:**
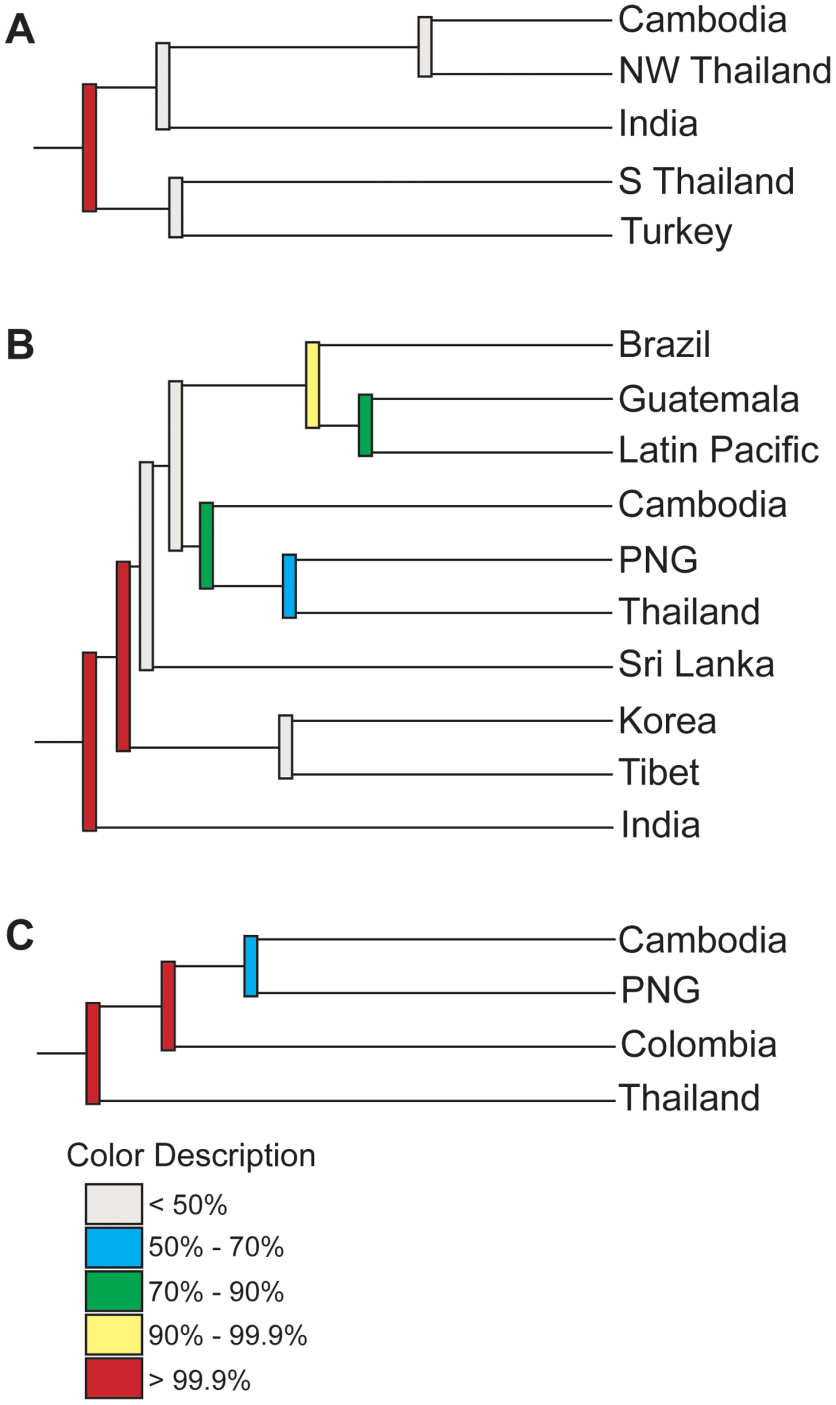
Jackknifed consensus trees demonstrate reproducible geographic clustering in *pvcsp* VK210 and VK247 isolates, but not *pvmsp-1*. The reproducibility of population clustering was assessed using 1000 jackknifed phylogenies. Individual populations clustered together or apart in each of the 1000 jackknifed phylogenies, and the frequency of a split between any two populations was quantified. Populations with grey bars (<50% splits) were genetically similar, while populations with red bars (>99.9% splits) were highly genetically distinct. Phylogenies were built from the *pvmsp-1* 42 kDa region (**A**), the *pvcsp* VK210 central repeat (**B**), and *pvcsp* VK247 central repeat (**C**).

To visualize whether 42 kDa sequences cluster according to geography, we compared all unique haplotypes in a single neighbor-joining tree, which revealed little clustering according to geographic origin (**Figure 4**). We quantified the extent of this clustering using Hudson's nearest-neighbor statistic (S_NN_), which assesses how frequently a variant's nearest neighbor is from the same population [51]. In both global and pairwise comparisons, *pvmsp-1* 42 kDa sequences from naturally evolving populations in Cambodia, India, and NW Thailand showed no evidence of strong geographic clustering (**Table 4**). To further confirm this finding, a neighbor-joining consensus tree was created and underwent 1000 jackknifed replicates (**Figure 5A**). Results showed that the predicted splits between most populations occurred only less than 50% of the time, providing strong evidence that there is minimal geographic clustering of *pvmsp-1* 42 kDa sequences.

**Table 4:** S_nn_ statistics for the *pvmsp-1* 42 kDa region and the *pvcsp* central repeat region.

*pvmsp-1* Global S_nn_ 0.410*	Cambodia	NW Thailand	S Thailand	India					
**Cambodia**									
**NW Thailand**	0.318								
**S Thailand**	0.780	0.865							
**India**	0.673	0.779*	0.865						
**Turkey**	0.897	0.946	0.750	0.917					

VK210 Global S_nn_ 0.517*	Cambodia	Thailand	India	Korea	Tibet	PNG	Sri Lanka	Brazil	Honduras
**Cambodia**									
**Thailand**	0.511								
**India**	0.824	0.778							
**Korea**	0.950*	0.92	0.977*						
**Tibet**	0.968*	1.000*	0.686	0.864					
**PNG**	0.7621	0.633	0.920*	0.923*	0.913				
**Sri Lanka**	1.000*	1.000*	0.955*	0.958*	0.926*	0.987*			
**Brazil**	0.844*	0.958*	0.943*	0.955*	0.969*	0.855	1.000*		
**Honduras**	0.824	1.000	0.976*	1.000	1.000*	1.000*	1.000*	0.881	
**Guatemala**	1.000*	0.933	0.970*	1.000	0.957*	0.929	0.960*	0.957*	0.708

VK247 Global S_nn_ 0.872*	Cambodia	Thailand	PNG						
**Cambodia**									
**Thailand**	1.000*								
**PNG**	0.904*	0.939*							
**Columbia**	1.000*	0.981*	0.935*						

S_nn_ values approaching 1 indicate genetic isolation while values near 0.5 indicate that two geographically disparate populations may approximate panmixia. Global and pairwise S_nn_ values show stronger geographic clustering among *pvcsp* VK210 and VK247 repeats than among *pvmsp-1* 42 kDa regions.

* indicates significance to (*p*≤0.05) after Bonferroni correction for multiple comparisons.

**Figure 6:**
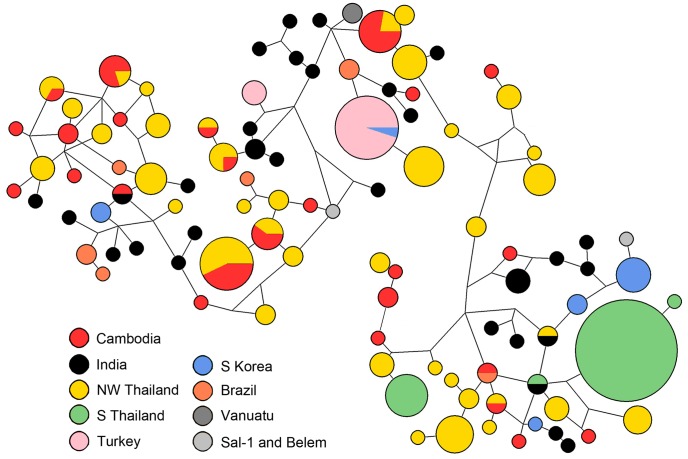
Median-joining network of diverse *pvmsp-1* populations proposes multiple mutational paths between geographically diverse populations. 286 *pvmsp-1* 42 kDa sequences from diverse geographical regions were used as input to create an unrooted median-joining network. This network is a visual representation of the mutational paths that may explain the observed sequence diversity. Each node represents an allele, node size represents the frequency of that allele (range n = 1 to n = 54), and node color corresponds to country of origin. Cycles within the diagram represent alternative evolutionary pathways. Corners represent obligate intermediate sequences that were not observed among the sampled alleles. Line length is not proportional to genetic distance.

To better understand the evolutionary relationships between *pvmsp-1* haplotypes from around the world, we employed a median-joining network to describe the set of potential mutational paths between all available global *pvmsp-1* 42 kDa sequences [53]. The network shows extensive admixture of parasite populations from diverse locales, with numerous mutational paths connecting haplotypes (**Figure 6**). With the exception of populations from S Thailand and Turkey, which have undergone recent bottlenecks, these data provide further evidence that there is no clustering by geography.

### *pvcsp* sequences

We sequenced the complete *pvcsp* gene from 43 isolates using the PacBio and Illumina platforms. *de novo* assembly of the Illumina paired-end short reads was not possible, due to over-collapse in the central repeat (CR) region, resulting in inappropriately short CRs. In contrast, PacBio long reads allowed the gene to be sequenced in its entirety and, after clustering, predicted 47 *pvcsp* haplotypes within the 43 samples. Reported error rates for PacBio sequencing have been high, especially for indels [63]; however, the use of Circular Consensus Sequencing allows single DNA fragments to be read multiple times, decreasing the error rate of the final predicted sequence. To check the accuracy of PacBio *pvcsp* haplotypes, individual haplotypes were used as a template for alignment of Illumina reads from the same clinical isolate. The addition of Illumina reads corrected only a single 1-bp deletion in a single haplotype. Therefore, after clustering, PacBio-predicted haplotypes have an error rate of 1/(∼1200 basepairs/sequence ×47 sequences), or approximately 0.002%.

Considering the entire gene, there were 24 unique haplotypes at the nucleotide level, and most genetic diversity was within the CR (**Figure 1**). Both nonapeptide repeat array types – VK210 (total n = 32, range 17–21 repeat units) and VK247 (total n = 15, range 20–21 repeat units) – were represented in our Cambodian population, with no VK210–VK247 hybrids (reviewed in [64]). The average Illumina short-read depth for each isolate was >1000, with all bases having ≥5 reads of coverage. In addition to our isolates, we identified one cohort of nearly complete *pvcsp* sequences (n = 27), and 12 cohorts of CR sequences (n = 385) [65]–[70] (**Table 1**). An extrapolated rarefaction curve showed that we sampled more than two thirds of the *pvcsp* CR haplotypes in this part of Cambodia, and that there are significantly fewer *pvcsp* CR variants in this region of Cambodia than *pvmsp-1* 42 kDa variants (**Figure 2**).

### Detecting signatures of selection within *pvcsp*

In contrast to *pvmsp-1*, the 5′ and 3′ non-repeat regions of *pvcsp* had no significant signatures of selection either by the MK test (data not shown) or Tajima's D test (**Table 1**). The 5′ non-repeat region in the Cambodian cohort showed a non-significant signature of balancing selection (**Table 1** **and** **Figure 3D**), which was due to a G38N amino acid polymorphism. This polymorphism also was observed in 6/16 parasites from the Latin Pacific region (JQ511263-JQ511276, JQ511279, JQ511286) and 2/27 parasites from Colombia (GU339072 and GU339085). The 3′ non-repeat region had little evidence of balancing selection, with Tajima's D values ∼0 (**Table 1** **and** **Figure 3D**). Within *pvcsp*, an 18 amino-acid C-terminal motif known as Region II (amino acid residues 311–328 in Sal1) is important for parasite invasion of hepatocytes [71] and purportedly contains both B and T-cell epitopes [72], [73]. Among all Cambodia and Colombia parasite isolates, this motif is completely conserved at the nucleotide and protein level, with an amino-acid sequence of EWTPCSVTCGVGVRVRRR, similar to previous reports [61].

To better understand the selective forces acting upon the *pvcsp* CR, we assessed the dN/dS ratio for Cambodian VK210 and VK247 [66]. Strikingly, synonymous substitutions were strongly favored in both VK210 (dN/dS = 0.267; Z test *p*<0.001) and VK247 (dN/dS = 0.166; Z test *p*<0.001) repeats. This is consistent with the finding that VK210 and VK247 isolates from around the world consistently demonstrate a depressed dN/dS ratio, suggesting that the VK210 and VK247 repeat regions are both under strong purifying selection [66].

The CR of *P. falciparum csp* is thought to evolve by slipped-strand mispairing [42]. To understand if a similar mechanism works in the *pvcsp* repeats, we studied the mismatch distribution of pairwise genetic distances between untranslated repeat units within each VK210 and VK247 repeat array type in Cambodia. Consistent with another study [68], we observed a strong right skew in the proportion of genetic differences between pairwise VK210 repeat comparisons, and between pairwise VK247 repeat comparisons, evidence that *pvcsp* repeats have a high proportion of identical or nearly identical repeats (data not shown). This finding is consistent with a continuous and rapid expansion and contraction of repeats by slipped-strand mispairing, which may be a mechanism to evade host immunity [42].

### Geospatial genetic diversity at the *pvcsp* central repeat

A recent study assessed global genetic diversity in the *pvcsp* CR, but did not define the correlates of differentiation between populations [66]. Moreover, this report investigated CR diversity by using a subset of the repeat region that was invariant in length. This approach may not reflect true population structure as it only assesses repeats early in the CR. Indeed, we have found that certain repeat types do cluster in locations within the repeat arrays (data not shown).

**Figure 7:**
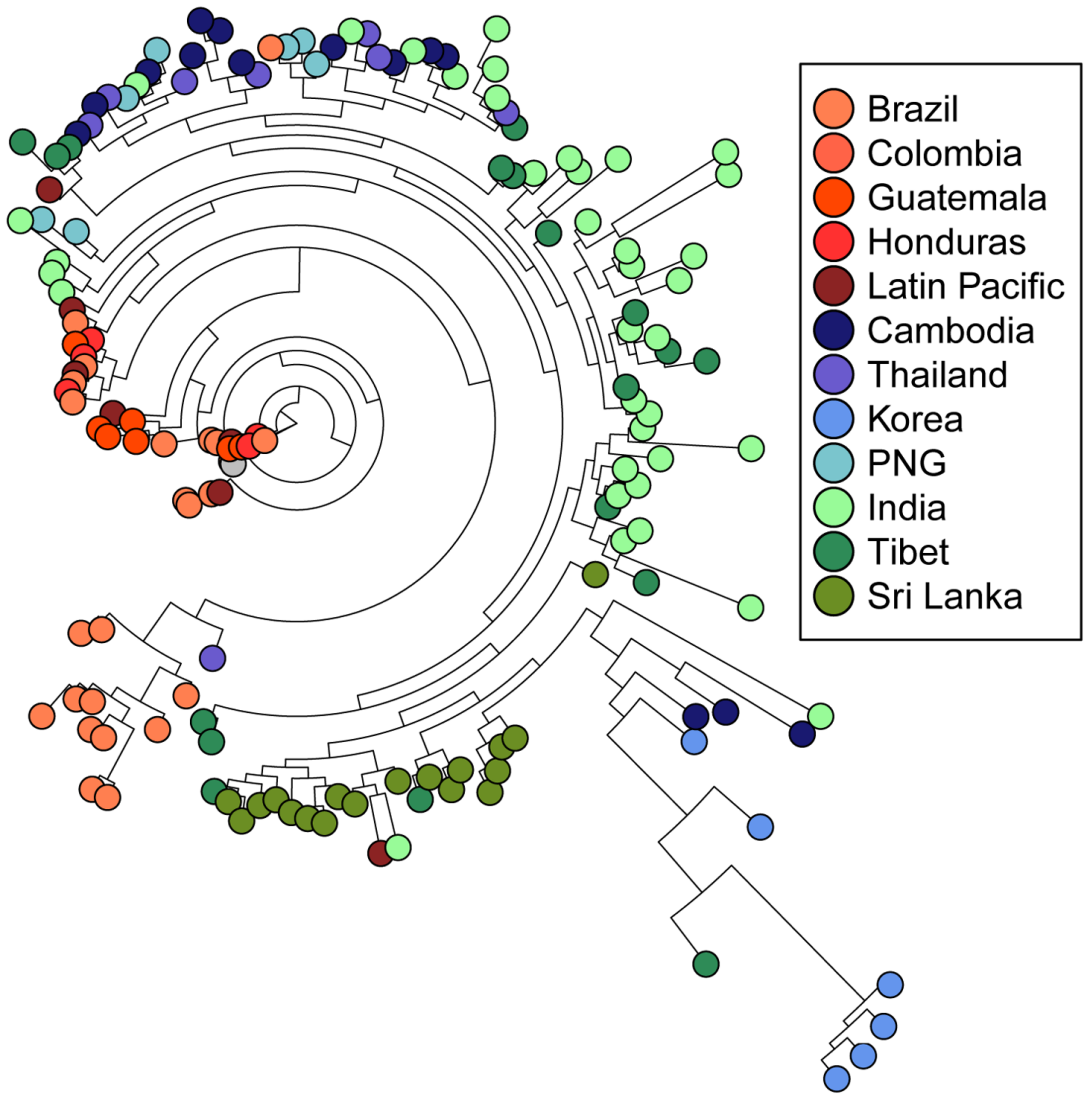
Neighbor-joining tree of *pvcsp* VK210 repeat arrays. All unique repeat array haplotypes from each *pvcsp* VK210 population set were plotted on a single unrooted, neighbor-joining phylogenetic tree. Visual inspection reveals strong geographic clustering by region and country. Latin American sequences are in shades of red, South East Asian sequences are in shades of blue, and South and Central Asian sequences are in shades of green.

To more rigorously study the global diversity of the *pvcsp* CR, we modeled CR repeat expansion, contraction, and substitution using MS_Align, which calculates an event-based genetic distance between CR haplotypes [46]. From these data, we constructed neighbor-joining trees for global VK210 and VK247 repeat arrays isolates (**Figures 7** **–** **8**). In contrast to *pvmsp-1*, the VK210 and VK247 trees revealed striking geographic clustering by country and continent. We quantified clustering using Hudson's S_NN_, and observed strong genetic differentiation between most geographically diverse parasite populations, in contrast to *pvmsp-1* (**Table 4**). To confirm this finding, neighbor-joining consensus trees for both VK210 and VK247 were subjected to 1000 jackknife replicates and the reproducibility of predicted splits between populations was tested demonstrating a strong correlation between genetic distance and geography (**Figure 5B–C**).

**Figure 8:**
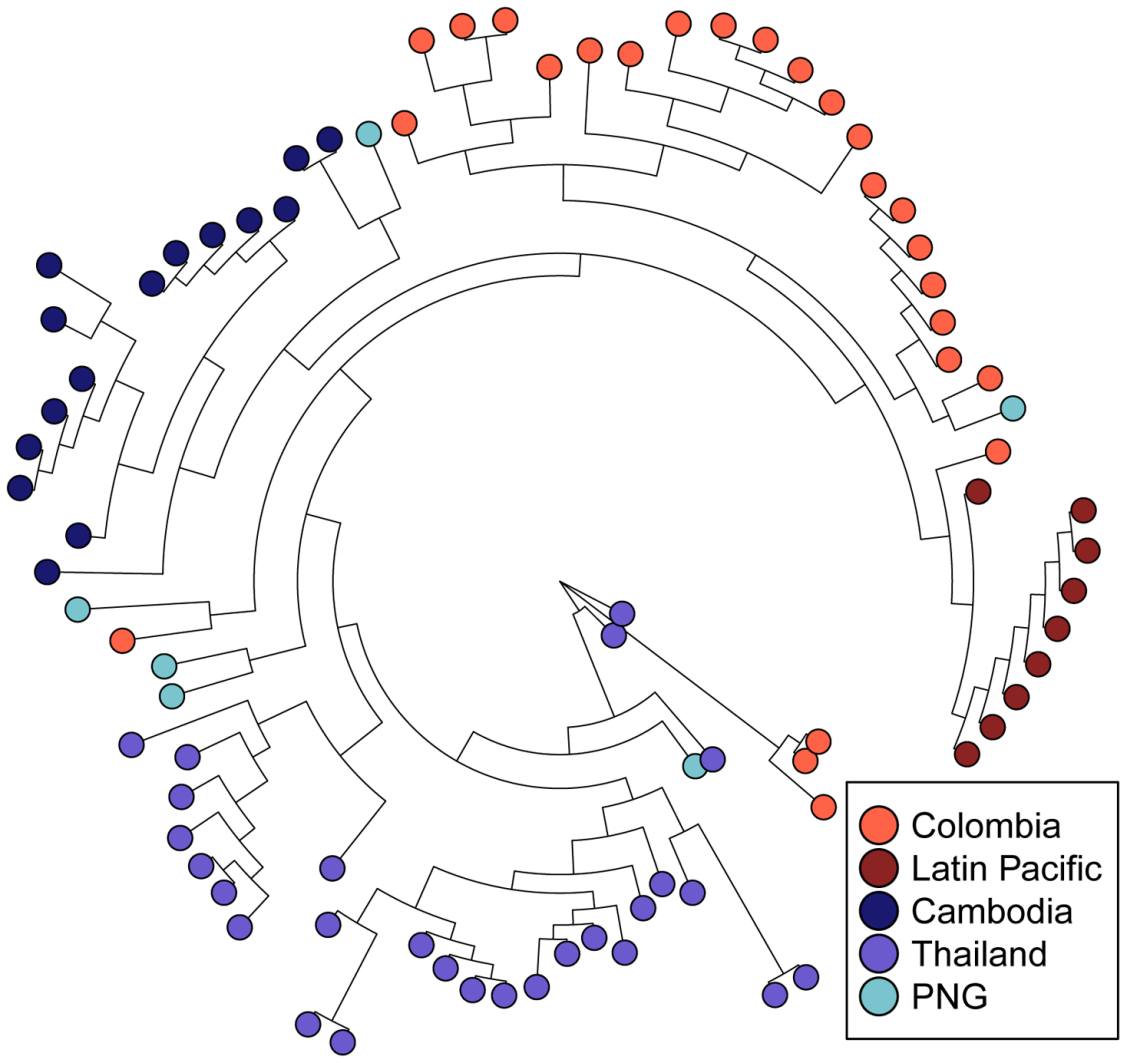
Neighbor-joining tree of *pvcsp* VK247 repeat arrays. All repeat array haplotypes from each *pvcsp* VK247 population set were plotted on a single unrooted, neighbor-joining phylogenetic tree. Latin American sequences are in shades of red, South East Asian sequences are in shades of blue.

We were able to define the peptide sequence basis of the clustering observed among *pvcsp* CR repeats. For VK210 repeats, almost all (81/84) Latin American repeat arrays contained either a 5′ (GDRADGQPA)_4_ or an internal (GDRADGQPA)_3–4_, while very few (11/278) of the Asian sequences contained one or both of these features. Similarly, for VK247 repeat arrays, all (34/34) Latin American sequences began with a single EDGAGDQPG repeat, while only one (1/44) Asian sequence began with this repeat. These sequence features may represent a reliable method to assign sequences to a geographic region.

## Discussion

This study (1) presents the first population set of *pvmsp-1* and *pvcsp* sequences from Cambodia, (2) identifies a signature of putative immune-mediated, frequency-dependent selection in the *pvmsp-1* 42 kDa region and the *pvcsp* CR, and (3) provides the most comprehensive evaluation to date of geospatial genetic diversity for these genes. We also demonstrate the feasibility of using a next-generation sequencing approach to study the genetic diversity of malaria antigens.

A distinguishing feature of this study is the use of next-generation sequencing methods to generate *P. vivax* amplicon sequence data from clinical isolates. This work represents a first step into this largely unexplored territory. As a relatively new technology, next-generation sequencing methods must be validated before use in molecular epidemiological studies. We provide evidence that the dominant Illumina-predicted *pvmsp-1* haplotypes are consistent with Sanger sequencing, and are fit for comparison with population sets generated by traditional sequencing methods. Methods for predicting multiple haplotypes from short-read sequencing are under development and will need further validation. We also demonstrate the ability of combined PacBio-Illumina haplotypes to predict *pvcsp* VK210 and VK247 haplotypes out of individual mixed infections. As next-generation sequencing methods are utilized more frequently for population genetic studies of infectious diseases, the methods introduced here will be further improved and will help to provide greater insight into *Plasmodia* population genetics.

### Evidence of selection in both *pvmsp-1* and *pvcsp*

We found compelling genetic evidence that the *pvmsp-1* 42 kDa intervening region is under strong immune pressure in multiple panmictic populations. Results from the MK test suggested that this region is under sustained selective pressure (**Table 2**); however, because a positive MK test can signify balancing selection or weak negative selection [74], [75], we tested the hypothesis that this region is under balancing selection using Tajima's D test of neutrality. Since multiple populations showed strong evidence of balancing selection by Tajima's D (**Table 1**, **Figure 3B**), we conclude that the intervening region is undergoing continual diversifying, balancing selection. An alternative hypothesis is that the positive Tajima's D values are an artifact of recent population contractions. Because (1) a positive Tajima's D was observed in multiple populations, and (2) other regions of *pvmsp-1* contained negative Tajima's D values, we conclude that the 42 kDa intervening region of *pvmsp-1* undergoes frequency-dependent (and likely immune-mediated) balancing selection.

Because PvMSP-1 is a merozoite surface antigen, it is highly accessible to antibodies and complement. The predicted structure of the 42 kDa region shows that the 33 kDa fragment covers the 19 kDa fragment [11], [76], limiting its exposure to the human immune system relative to the 33 kDa fragment. This observation could explain the extensive balancing selection present in the 33 kDa fragment (specifically, the intervening region) but not in the 19 kDa fragment. Additionally, this finding suggests that the sliding window approach for evaluating polymorphism and balancing selection may help generate hypotheses about functionally important (19 kDa fragment, for example) or immunologically dominant (the intervening region, for example) regions of *P. vivax* proteins.

For *pvmsp-1*, Tajima's D and *F*_ST_ were inversely correlated. Populations with strong evidence of high Tajima's D in the *pvmsp-1* intervening region showed a low genetic differentiation by *F*_ST_. This suggests that in naturally evolving populations, diversification of this region is extensive and maintains a similar range of genetic diversity despite geographic distance. Populations that have undergone a recent bottleneck show a low Tajima's D with relatively few variants and strong genetic differentiation from more diverse populations. This suggests that if strain-specific immune responses are important in vaccine efficacy, vaccines may work more effectively if other interventions can be used to bottleneck the population, thus decreasing its genetic diversity [54].

The central repeat region (CR) is a primary immunodominant region of PvCSP. Though alignment-based methods to assess for selection (Tajima's D, for example) cannot be employed in a tandem repeat region, there is wide-ranging evidence that selective pressures shape the genetic composition of the *pvcsp* CR [77]–[81], including new evidence hinting that hosts develop strain-specific immunity to *P. falciparum* NANP repeats of varying lengths [82]. Indeed, the presence of two distinct repeat types (VK210 and VK247) may itself be evidence of selection as suggested in a study of the *P. cynomolgi csp* CR [80].

Our analysis of the two CR array types, VK210 and VK247, also suggests that selection is occurring in this region. In pairwise comparisons of nucleotide and amino acid differences we observed a positive skew showing decreased differences among repeat units. This finding is consistent with Patil et al.'s study of *pvcsp* isolates from Brazil [68], and provides further evidence that both VK210 and VK247 repeat arrays may continuously evolve via slipped-strand mispairing [42]. Furthermore, consistent with a recent study of selection in worldwide *pvcsp* isolates [66], we found that Cambodian *pvcsp* VK210 and VK247 isolates have a strong bias toward synonymous substitutions. This signature of purifying selection is consistent with reports from *pfcsp* [83]–[85] and suggests that there are a limited number of amino acid polymorphisms allowable within this repeat region. Taken together, these findings suggest that expansion, contraction, and rearrangement of repeat units, rather than generation of novel repeat units through mutation, maintain genetic diversity at the *pvcsp* locus in both VK210 and VK247 variants. This phenomenon may be responsible for immune evasion [68], [86].

Although these two vivax genes are orthologs of well-characterized vaccine candidate antigens from *P. falciparum* malaria, substantial differences are seen in the effects of immune selection between these genes and their orthologs. Previous reports have shown that the functionally similar *pfmsp-1* 42 kDa fragment has relatively low nucleotide diversity and lacks evidence of balancing selection by Tajima's D [87]. *pfcsp*, on the other hand, shows a high level of nucleotide diversity [88]–[90] and modest Tajima's D elevations in the C-terminal T cell epitopes [88], [91]. These patterns are in stark contrast to our observations in *P. vivax*, and this highlights the need for *P. vivax*-specific studies to determine appropriate candidate vaccine antigens.

Finally, our analysis of the *pvmsp-1* 42 kDa region underscores the importance of selecting an appropriate parasite population for population-genetic studies. We did not observe signatures of balancing selection in *pvmsp-1* populations from S Thailand or Turkey. This is likely due to bottlenecks secondary to robust malaria control measures employed in S Thailand [54] and limited human migration in Turkey [58]. Thus, appropriate selection of panmictic populations for these studies is critical.

### Differing patterns of geospatial genetic diversity at *pvmsp-1* and *pvcsp*

Using both tree-based and statistical methods [92], we found that *pvcsp*, but not *pvmsp-1*, showed strong clustering by geography (**Tables 3** **–** **4** **and** **Figures 4** **–** **8**). For *pvmsp-1*, we observed little geographic clustering among naturally evolving parasite populations, suggesting that immune selection maintains similar *pvmsp-1* alleles around the globe. Notably similar findings have been described in Duffy Binding Protein and Thrombospondin-related anonymous protein in vivax malaria [61], while a recent global survey of diversity in the Apical Membrane Antigen 1 found evidence of geographically restricted haplotypes [93]. In contrast to *pvmsp-1*, we found that *pvcsp* variants demonstrate strong evidence of geographic clustering. This juxtaposition between *pvmsp-1* and *pvcsp* sequences is similar to what has previously been described for merozoite and sporozoite antigens in *P. falciparum* [94]. The population sets included in this survey were collected in different years. While it is known that novel *P. vivax* surface antigen types can appear in the course of a decade [57], it is difficult to assess the magnitude of this effect on our analyses. As more *pvmsp-1* and *pvcsp* population sets are collected, this will become clearer.

It is interesting that the CR of *pvcsp* shows evidence of multiple forms of selection: (1) the depressed number of non-synonymous mutations suggests purifying selection, (2) the differences in CR genotypes between geographic locations suggests directional selection, and (3) the genetic composition of the repeats suggests rapid expansion and contraction, possibly due to immune selection. It is unclear what drives the first two signatures of selection. We hypothesize a model in which purifying selection within a population limits the amino acid composition of repeats due to functional constraints of the protein, while directional selection between populations is driven by environmental factors.

One environmental factor that may explain both the purifying and directional selection of parasite *pvcsp* CR sequences is the mosquito vector. The circumsporozoite protein is expressed in the mosquito during oocyst development [95] and in the salivary glands [96], [97]. It is also critical in sporozoite motility [15]. We found no overlap in the distribution of Anopheline species between the countries from Asia and Latin America included in this study (data not shown) [98]–[100]. Furthermore, there is substantial evidence that different Anopheline species and strains show differential ability to be infected by malaria [101]–[104].

Regardless of the cause of the differing patterns of geospatial genetic diversity we observed in *pvmsp-1* and *pvcsp*, the observation itself has significance for vaccine design. The malaria vaccine field is just beginning to unravel how antigenic diversity within a single parasite population can reduce vaccine efficacy [105]. Our findings highlight an additional level of complexity that will hinder the implementation of a vivax vaccine – antigenic variability. While the effects of immune cross-reactivity against different antigenic variants aren't fully known, the extensive intrapopulation variability seen in *pvmsp-1* may necessitate a highly multivalent *pvmsp-1* vaccine, while the dramatic interpopulation variability seen in *pvcsp* suggests that a PvCSP-based vaccine that is effective in one part of the globe may not be effective in other regions. Thus, a thorough understanding of the geospatial genetic diversity of candidate vaccine antigens must inform antigen selection for vaccine design.

### GenBank accession numbers

*pvcsp* sequences: JX461243-JX461285 and KJ173797- KJ173802

*pvmsp-1* sequences: JX461286-JX461333

## Supporting Information

Figure S1 **Geographic distribution of** ***P. vivax*** **populations contributing to this study.** In total, we identified 13 populations with *pvmsp-1* 42 kDa fragment sequences and 13 populations with *pvcsp* central repeat or whole-gene sequences. These populations were collected from 14 countries, pictured above. For countries with n≥10 isolates, the total number of *pvmsp-1* and *pvcsp* isolates is marked.
(TIF)

Figure S2 ***F*** **_ST_ values at polymorphic sites within the** ***pvmsp-1*** **42 kDa intervening region.** Available parasite populations with n>25 individuals (Cambodia, India, NW Thailand, S Thailand, and Turkey) share 42 variable sites within the 42 kDa intervening region of *pvmsp-1*. *F*_ST_ values for each variable site were calculated in a pairwise manner between all five populations. *F*_ST_ values approaching 0 indicate limited inter-population variability at that site, while values approaching 1 indicate substantial inter-population variability. Coordinates are reported for every third polymorphic site.
(TIF)

Table S1 ***pvmsp-1*** **and** ***pvcsp*** **sequences included in this study.** The PlasmoDB gene identifier is PVX_099980 for *pvmsp-1* and PVX_119355 for *pvcsp*. *Indicates the year the sequences were made available in GenBank. ^†^Indicates study unpublished but sequences available in GenBank.
(DOCX)
