# Supplementary figures and images for: Differing Patterns of Selection and Geospatial Genetic Diversity within Two Leading Plasmodium vivax Candidate Vaccine Antigens
Source: PLoS Negl Trop Dis. 2014 Apr 17;8(4):e2796. doi: 10.1371/journal.pntd.0002796 (PMC3990511; doi:10.1371/journal.pntd.0002796)

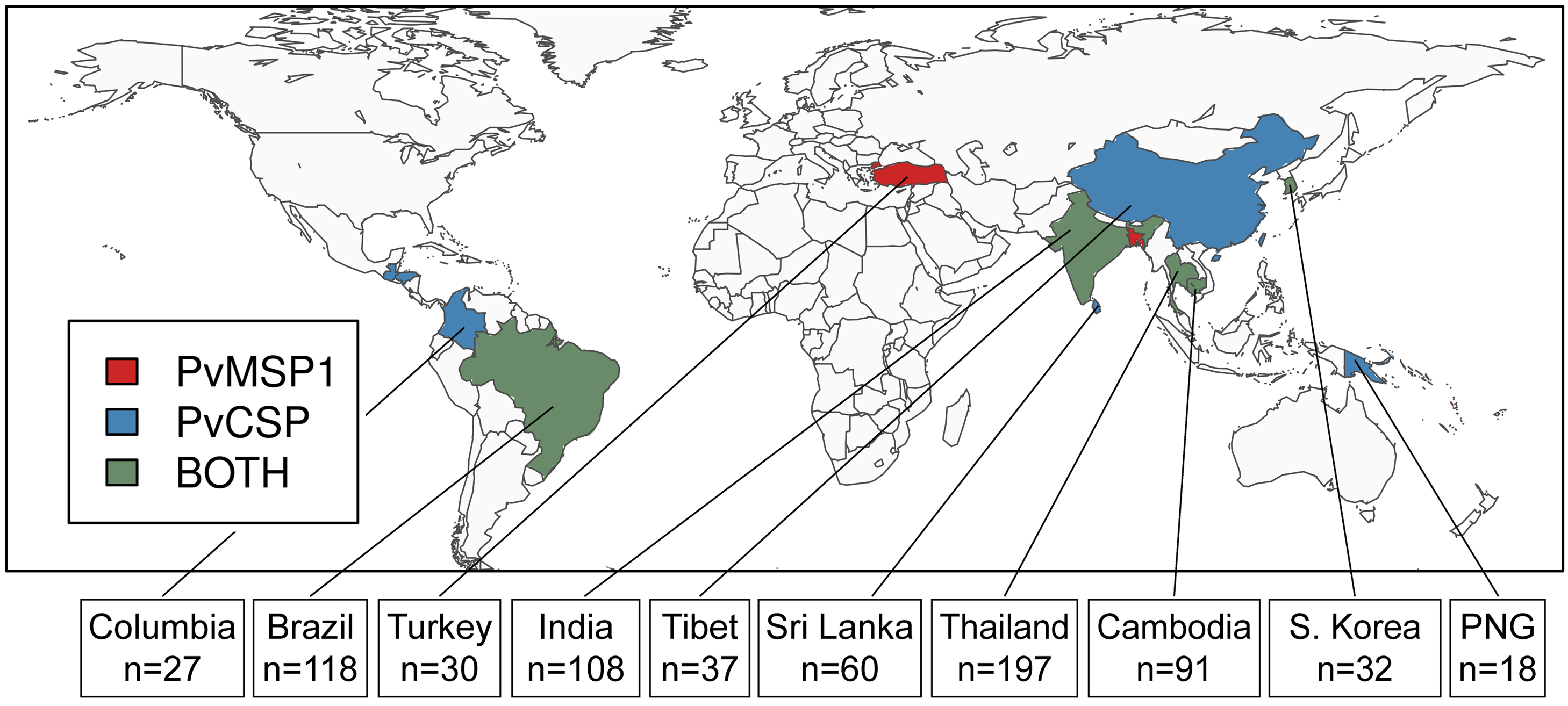

Supplement: Figure S1 — Geographic distribution of P. vivax populations contributing to this study. In total, we identified 13 populations with pvmsp-1 42 kDa fragment sequences and 13 populations with pvcsp central repeat or whole-gene sequences. These populations were collected from 14 countries, pictured above. For countries with n≥10 isolates, the total number of pvmsp-1 and pvcsp isolates is marked. (TIF) [file pntd.0002796.s001.tif]

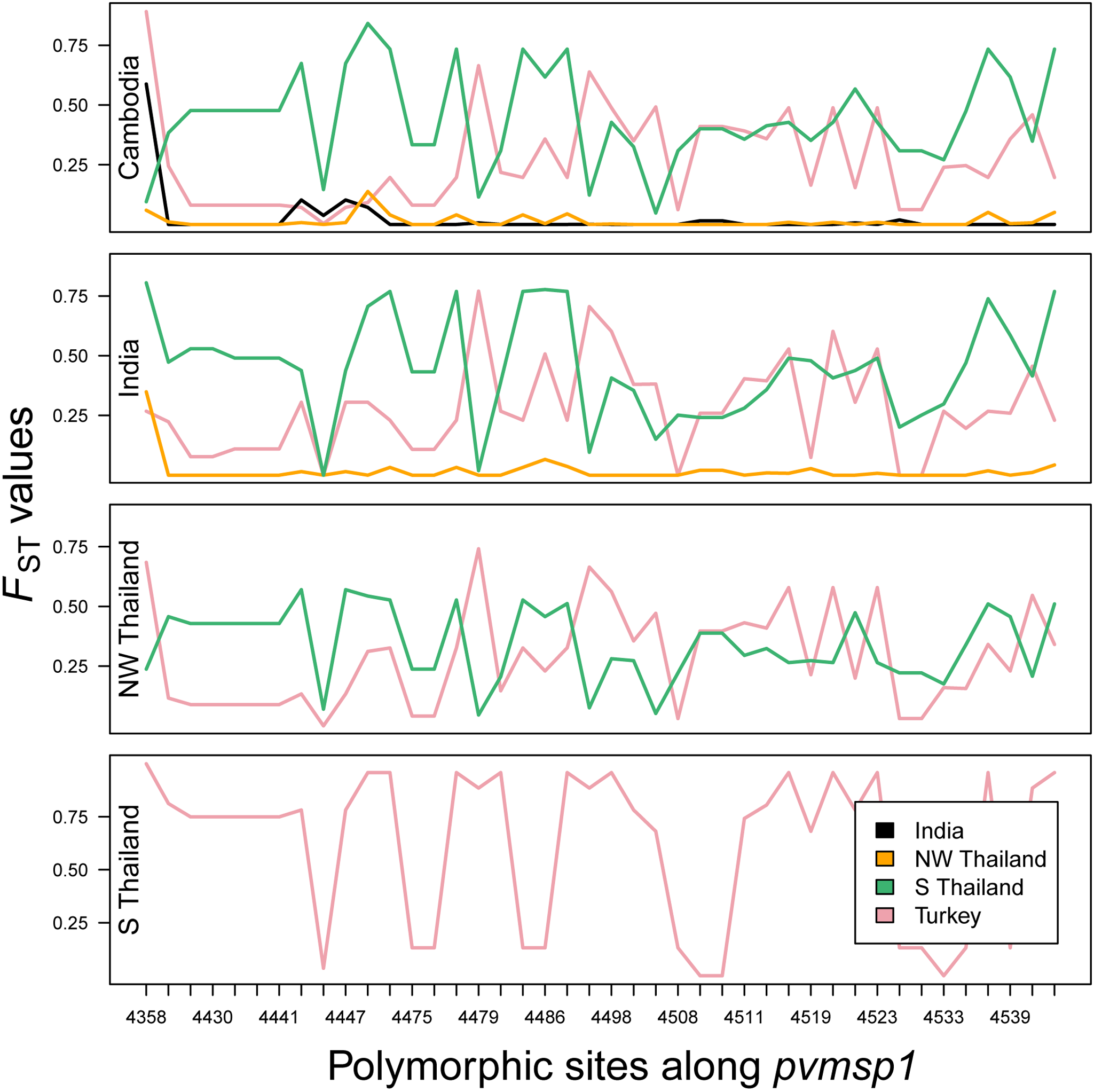

Supplement: Figure S2 — F ST values at polymorphic sites within the pvmsp-1 42 kDa intervening region. Available parasite populations with n>25 individuals (Cambodia, India, NW Thailand, S Thailand, and Turkey) share 42 variable sites within the 42 kDa intervening region of pvmsp-1. F ST values for each variable site were calculated in a pairwise manner between all five populations. F ST values approaching 0 indicate limited inter-population variability at that site, while values approaching 1 indicate substantial inter-population variability. Coordinates are reported for every third polymorphic site. (TIF) [file pntd.0002796.s002.tif]
