# Supplementary material for: Differing Patterns of Selection and Geospatial Genetic Diversity within Two Leading Plasmodium vivax Candidate Vaccine Antigens
Source: PLoS Negl Trop Dis. 2014 Apr 17;8(4):e2796. doi: 10.1371/journal.pntd.0002796 (PMC3990511; doi:10.1371/journal.pntd.0002796)
Supplement: Table S1 — pvmsp-1 and pvcsp sequences included in this study. The PlasmoDB gene identifier is PVX_099980 for pvmsp-1 and PVX_119355 for pvcsp. *Indicates the year the sequences were made available in GenBank. †Indicates study unpublished but sequences available in GenBank. (DOCX) [file pntd.0002796.s003.docx]

| **Gene** | **Region** | **Country** | **Year** | **Loci Included** | **N** | **Accession No.** | **Reference** |
| --- | --- | --- | --- | --- | --- | --- | --- |
| ***pvmsp-1*** | SE Asia | Cambodia | 2006 - 2007 | 42 kDa region | 44 | JX461286-JX461333 | Present Study |
|  | SE Asia | NW Thailand | 2006 - 2007 | Whole gene | 65 | GQ890872 - GQ890916; AF435595 - AF435615 | Jongwutiwes2010 |
|  | SE Asia | S Thailand | 2006 - 2007 | Whole gene | 67 | GQ890975 - GQ891041 | Jongwutiwes2010 |
|  | SE Asia | N Thailand | 1997 - 1998 | Whole gene | 19 | AF435595 - AF435614 | Putaporntip 2002 |
|  | SE Asia | Bangkok | 1997 | Whole gene | 1 | AF435615 | Putaporntip 2002 |
|  | Asia Pacific | Vanuatu | 1996, 1998 | Whole gene | 2 | AF435632, AF435634 | Putaporntip 2002 |
|  | S Asia | India | 2004 - 2007 | 42 kDa | 28 | EU430452 – EU430479 | Thakur 2008 |
|  | S Asia | India | 1999 | Whole gene | 1 | AF435639 | Putaporntip 2002 |
|  | S Asia | Bangladesh | 1994 | Whole gene | 5 | AF435616 - AF435620 | Putaporntip 2002 |
|  | E Asia | S. Korea | 1996 - 2009 | Whole gene | 8 | HQ171934 - HQ171941 | Han 2011 |
|  | E Asia | S. Korea | 1998 | Whole gene | 4 | AF435635 - AF435638 | Putaporntip 2002 |
|  | W Asia | Turkey | 2007 - 2008 | Whole gene | 30 | AB564559 - AB564588 | Zeyrek 2010 |
|  | S America | NW. Brazil | 1995, 1997 | Whole gene | 8 | AF435622 - AF435631 | Putaporntip 2002 |
| ***pvcsp*** | Asia Pacific | Cambodia | 2006 - 2007 | Whole gene | 47 | JX461243-JX461285, KJ173797- KJ173802 | Present Study |
|  | Asia Pacific | Thailand | 2009* | CR | 45 | HQ011279 - HQ011323 | N/A |
|  | S Asia | India | 2008* | CR | 79 | FJ491063 – FJ491141 | N/A |
|  | E Asia | Korea | 2006* | CR | 20 | DQ859736 – DQ859767 | N/A |
|  | E Asia | Tibet | 2008* | CR | 37 | FJ601724 - FJ601761 | N/A |
|  | Oceania | Papua New Guinea | 1999, 2001 - 2003 | CR | 18 | EU031819 - EU031836 | Henry-Halldin 2011 |
|  | S Asia | Sri Lanka | 1998-2000 | CR | 60 | [JQ362595](https://www.ncbi.nlm.nih.gov/nuccore/JQ362595.1) - [JQ362654](https://www.ncbi.nlm.nih.gov/nuccore/JQ362654.1) | Dias 2013 |
|  | S America | Columbia | 2009 | Whole gene | 27 | GU339059 - GU339086 | Hernandez-Martinez 2011 |
|  | S America | Brazil | 2004 - 2005 | CR | 45 | FJ845383 – FJ845391 | Patil 2010 |
|  | S America | Guatemala | 2012* | CR | 7 | [KC154040](https://www.ncbi.nlm.nih.gov/nuccore/KC154040.1) - [KC154046](https://www.ncbi.nlm.nih.gov/nuccore/KC154046.1) | Mendizabal-Cabrera^†^ |
|  | S America | Latin Pacific | 2012* | CR | 24 | [JQ511263](https://www.ncbi.nlm.nih.gov/nuccore/JQ511263.1) - JQ511286 | Gonzalez-Ceron^†^ |
|  | S America | Brazil | 1997 - 1999 | CR | 41 | [DQ978649](https://www.ncbi.nlm.nih.gov/nuccore/DQ978649.1) - [DQ978689](https://www.ncbi.nlm.nih.gov/nuccore/DQ978689.1) | Santos-Ciminera 2007 |
|  | S America | Honduras | 2010-2011 | CR | 9 | [JQ903593](https://www.ncbi.nlm.nih.gov/nuccore/JQ903593.1) - [JQ903601](https://www.ncbi.nlm.nih.gov/nuccore/JQ903601.1) | Lopez 2012 |

**Table S1. *pvmsp-1* and *pvcsp* sequences included in this study.** The PlasmoDB gene identifier is PVX_099980 for *pvmsp-1* and PVX_119355 for *pvcsp*. *Indicates the year the sequences were made available in GenBank. ^†^Indicates study unpublished but sequences available in GenBank.
